# Copper sulfide nanoparticles application under field condition increases *Eruca sativa* morpho-physiological response, yield, and biodiesel production for sustainable energy

**DOI:** 10.1038/s41598-025-20523-7

**Published:** 2025-10-21

**Authors:** Jehangir Khan, Mah Rukh, Mushtaq Ahmad, Muhammad Zia

**Affiliations:** 1 Department of Biotechnology, Quaid-i-Azam University, Islamabad, 45320 Pakistan; 2 Department of Biosciences, University of Wah, Quaid Avenue Wah Cantt, Wah, 47040 Pakistan; 3 Department of Plant Sciences, Quaid-i-Azam University, Islamabad, 45320 Pakistan

**Keywords:** GC-MS, FT-IR, NMR, NPs, Oil content, Fuel properties, Biochemistry, Environmental sciences, Nanoscience and technology

## Abstract

Agronomic parameters have been extensively studied under lab conditions to determine effect of nanoparticles (NPs). However, the yield characteristics have not been determined especially to biodiesel producing crops. This study explores the effect of copper sulfide (CuS) NPs on the growth, biochemical properties, productivity, and oil content of *Eruca sativa* under field condition. The oil extracted from *E. sativa* seeds was converted to biodiesel via transesterification reaction and analyzed through FT-IR, GC-MS and NMR. Further the fuel properties were compared with international standards. The results demonstrate that CuS NPs markedly improved root (20.23%) and shoot length (21.17%), branches per plant (45.64%), plant biomass (49.46%), siliqua per plant (48.68%), seeds per siliqua, and oil percentage (18.27%) compared to control plants. Atomic absorption spectroscopy depicts that root accumulated maximum Cu followed by shoot and seeds. Biochemical analysis showed that plants treated with CuS NPs had higher level of protein (175.16 mg/kg FW), superoxide dismutase (0.63 nM/min/gFW) and peroxidase (0.74 nM/min/gFW) activities as compared to control plants. The roots and shoots of *E. sativa* showed enhanced total phenolic content, total flavonoid content, and improved antioxidant activities. The biodiesel yield was up to 85% using 1% KOH as catalyst and 6:1 oil to methanol ratio. FT-IR, NMR and GC-MS analyses of biodiesel indicate that CuS NPs has negligible effect on the biodiesel composition. Fuel properties i.e., pour point, cloud point, flash point, viscosity, density, sulfur content and ash content are found to comply with international standards. This study highlights the potential of CuS NPs in elevating agricultural yield and offers a sustainable approach to biofuel production.

## Introduction

The application of nanoparticles (NPs) in agriculture has emerged as novel strategy to promote plant growth productivity and resilience. Copper sulfide nanoparticles (CuS NPs), in particular, have been evaluated for their potential antimicrobial properties^1^ and their role in enhancing plants agronomic parameters. Yet, the impact of CuS NPs on *Eruca sativa* under field condition remains unexplored particularly regarding agronomic parameters and oil content characteristics. Copper (Cu) is an essential micronutrient that promotes plant growth, plays an important role in photosynthesis, protein metabolism, and electron transport chain^2–4^. Cu based NPs have been used as fertilizer, growth regulators, pesticides, and herbicides^5^. The size, high surface area, shape, and peculiar chemical and physical properties of NPs affect the plant growth and nutrition value^6^. Due to their slow ion release potential and higher adsorption, NPs including Cu NPs have gained attention in agricultural applications^7,8^. The bioaccumulation and translocation factors determine the uptake and translocation potential of metallic NPs and their respective ions in crops^9^. Cu nanoparticles have shown a positive effect on plant height, biomass, yield, and seed quality of *Glycine max*^8,10^. The positive effect on the growth and development of plants also include tolerance to biotic and abiotic variations, and stress mitigation due to biochemical and metabolic functions^11^. Under drought stress, Cu NPs increased total number of seeds, grain yield, chlorophyll content, and enzymes activities in maize^12^. Foliar application of Cu NPs increased fruit size in tomato^13^ and ameliorates toxicity of heavy metal by evoking antioxidant enzymes activities^14^. Rameen et al.^15^ reported improvement in productivity and oil quality of *Brassica napus* upon exposure to Cu NPs. Moreover, assessing the antioxidant capacities of the plant are important for understanding the defense mechanism of plants and its potential application in plant health. Sulfur (S) is an important component of S-containing amino acids and proteins. It promotes growth and metabolism of the plants, and regulates crop productivity and quality. Sulfur also minimizes the negative impact of heavy metals via higher antioxidant enzymes activities^16^.

*Eruca sativa* L. is a member of herbaceous family Brassicaceae (mustard family), commonly known as taramira, rocket salad, Rocket seed or arugula. It is a leafy vegetable, famous for its characteristic flavor, nutritional value, and oil content. *E. sativa* has also gained attention due to its rich phytochemical profile, particularly its phenolic and flavonoid compounds, which contribute to its antioxidant properties (^17,18^. These bioactive compounds associate with different health benefits, including antimicrobial and anti-inflammatory impacts^19^. The oil extracted from the seeds of *E. sativa* is a potential feedstock for biodiesel production. Biodiesel is a renewable energy source and may be used as alternative to conventional fossil fuel. Moreover, it is nontoxic, environment friendly, emit low gases, and require no modification for diesel engine^20^. Efficient and cost-effective production of biodiesel faces many challenges including high prices. Among 350 oil producing crops used as biodiesel feed stock, 95% are edible that led to food versus fuel conflict^21,22^.

The current study was aimed to evaluate the effect of copper sulfide nanoparticles (CuS NPs) on *Eruca sativa* under field conditions. Different agronomic parameters were measured. Plants extracts were assessed for antioxidant properties, and oil extracted from seeds was analyzed for biodiesel production. This inclusive approach peruses to explicate the multifaceted impact of Cu-based treatments on *Eruca sativa*, contributing significant insights into sustainable agricultural practices and biodiesel production.

## Materials and methods

The study describes synthesis, characterization of CuS NPs and their application as nanofertilizer under field condition to analyze growth, biochemical response, and yield characteristics of *E. sativa* plants. The seed oil was converted into biodiesel and characterized. The study was carried out at Quaid-i-Azam University Islamabad and National Agriculture Research Center (NARC) Islamabad. All the chemicals used in this study were purchased from Merck or Sigma-Aldrich otherwise mentioned.

### Synthesis and characterization of CuS NPs

Co-precipitation method as reported by Al-Jawad et al.^23^, was followed for the synthesis of CuS NPs. In short 0.5 M Na_2_S aquous solution was slowly mixed with 0.5 M CuSO_4_.5H_2_O solution. The reaction was stirred at 1500 rpm for 2.5 h. Thereafter, the color changed to greenish black. The CuS NPs were collected by centrifugation at 8000 rpm for 10 min at 25°C. The NPs were washed thrice with distilled water, dried, and calcinated at 500 °C for 2 h.

The morphology of NPs sample was determined through scanning electron microscopy (SEM, TESCAN MIRA 3) and images were recorded at different magnifications operating with a 20kV electron beam. The elemental analysis of CuS NPs was carried out by Energy-dispersive X-ray spectroscopy (EDX) at the energy range of 0-20 kV and current of 1 x 10^−9^ A. X-ray diffraction (XRD) was used to analyze crystallinity of NPs by Model D8, Bruker AXS. The XRD pattern was obtained by using *Cu Kα* radiation (λ = 1.5406); the 2θ range was from 20 to 80° at a scanning rate of 0.02 deg/sec., increment = 0.02, operating voltage = 40 kV, and operating current = 30 mA. The average size of CuS NPs was calculated using Debye-Scherrer equation.

**D = kλ/βcosθ**

Where D is the size of the crystallite, k is the crystal’s shape factor which is 0.9, λ is the wavelength of Cu Kα which is 1.5404 Å, θ is the Braggs angle of the diffraction peak and β is the angular full width at half maximum (FWHM) of diffraction peak, measured in radian.

The surface morphology of NPs for presence of functional groups was analyzed through Fourier transform infrared (FTIR) spectrometer (Nicolet^TM^380). The spectrum was acquired with a resolution of 2 cm^−1^ over the range 500–4000 cm^−1^.

### Description of experimental site

**Fig. 1:**
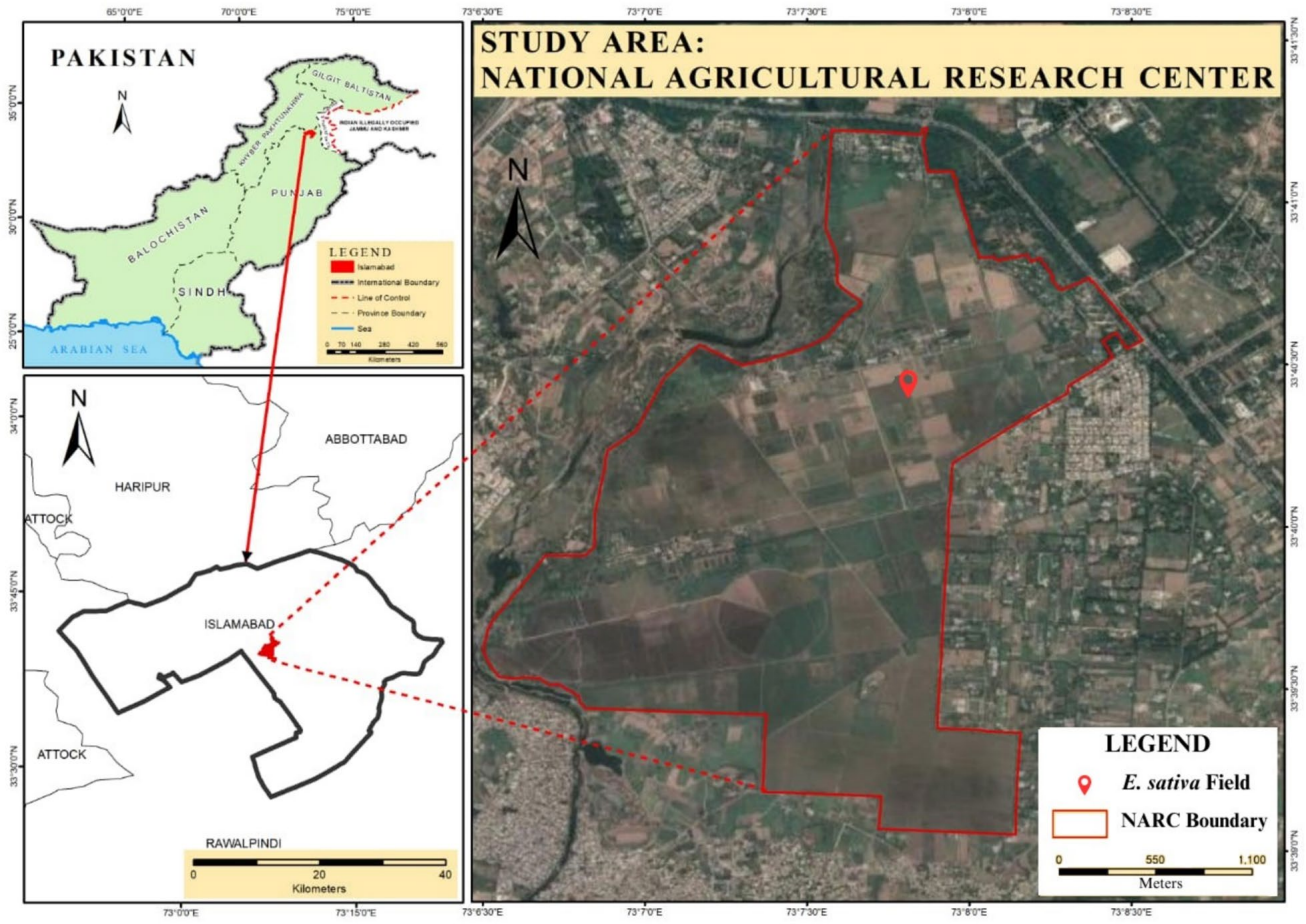
Study area map of NARC, Islamabad Pakistan. (Figure copied from^24^ after permission).

This study was conducted in open field at National Agriculture Research Centre (NARC) (33.4°N, and 73.8°E) Islamabad, Pakistan. The soil analysis before experiment, meteorological data during study, and study area map of NARC are shown in Table 1&Table 2, and Figure 1 respectively. According to Köppen climate classification, the experimental site is situated in a humid subtropical climate zone. Soybean, chickpea, sesame, linseed, and arugula etc. are the major crops of the selected study area. *Eruca sativa*, a rabi crop was cultivated in October-November 2022 followed by harvesting in April 2023.

**Table 1:** Physiochemical properties of the experimental soil before experiment.

EC (dS/m)	pH	NO_3_-N	P	K	Cu	Organic Matter %
		mg/kg				
0.11	7.84	0.67	4.72	138	21.4	0.73

**Table 2:** Metrological data of experimental site from October 2022 to April 2023.

**Month**	**Max Temp (°C)**	**Min** **Temp (°C)**	**Wind Speed. km/day**	**Pan Evap (mm)**	**Rainfall (mm) (rainy days)**	**Relative Humidity (%)**
October	30.03	14.35	28.48	3.10	11.97 (3)	81
November	23.77	8.63	19.28	1.61	10.71 (6)	90
December	20.68	4.13	20.56	1.27	8.47 (1)	47
January	16.61	3.26	26.51	1.18	7.58 (6)	56
February	23.11	6.93	31.01	2.13	3.74 (4)	51
March	24.32	10.77	33.07	2.69	9.48 (10)	59
April	28.63	14.43	45.77	4.37	7.01 (10)	47

### Field study

*Eruca sativa* (Var. PM-26187) seed were sown in a loamy soil (pH 7.73). A plot of 3.2 × 3.4 meters (10.88 m^2^) was selected for each treatment, the row length was kept 3.2 m, and the distance maintained between two beds was 2.30 m with a 0.70m path between the rows. After irrigation, the soil was ploughed and seed were sowed in the month of October 2022. The weeds were removed after 15 days of seed germination and the plant-to-plant distance was kept 4-6 cm by thinning. CuS NPs and CuSO_4_ were applied by broadcasting in soil at the rate of 8 Kg ha^−1^ at 6-8 leaf stage. Randomized Complete Block Design (RCBD) was used to conduct the study in triplicate. The nitrogen and potassium fertilizer (50 Kg/ha) were applied in the experimental plots and the field was irrigated when required. The crop was harvested in the month of April 2023. At harvesting stage, 10 plants were randomly picked from each treatment to measure selected agronomic parameters. Quantitative characteristics i.e., root and shoot length, total branches per plant, dry weight per plant, siliqua per plant, and seed per siliqua were recorded. The crop was harvested and seeds were manually isolated, dried, and stored for further analysis.

### Determination copper

The copper content in soil and plant parts (root, shoot, and seed) was measured by atomic absorption spectroscopy (AAS). The post-harvest soil was collected and after oven drying, 1 g soil was digested on a hot plate in fume hood at 80 °C in 15mL aqua regia till the mixture become transparent. The aqua regia was prepared by mixing 35% HCl and 70% high-purity HNO_3_ in a ratio of 3:1, respectively. The root, shoot, and seed were grinded in electric blender. Each sample (0.25g) was mixed in a freshly prepared acidic solution (HClO_4_, H_2_SO_4_, and HNO3 at a ratio of 0.5: 1: 5). The samples were digested until the solution become transparent and the final volumes was made 50 mL by distilled deionized water. After filtering the solution through Whatman filter paper 42, the filtrate was analyzed through atomic absorption spectrometer (PerkinElmer, Waltham, MA, USA) for the determination the amount of Cu in soil samples and plant material. The bioaccumulation factor (BAF) and translocation factor (TF) were calculated by using the following equations.

BAF = Concentration of Cu in plant tissues/Concentration of Cu in the soil

TF = Concentration of Cu in shoot and seeds/Concentration of Cu roots

### Estimation of total protein, SOD, and POD

Fresh leaves were collected at fruiting stage, rinsed with distilled water, chopped, and pasted in mortar pestle in pH 7 phosphate buffer. The transparent solution was centrifuged at 10,000 rpm for 10 min at 4°C. The supernatant was used for the estimation of total protein content, SOD, and POD activities. Total soluble protein in supernatant was measured followed the methodology reported by Lowry et al.^25^.

For determination of superoxide dismutase (SOD) activity, the method reported by Ullah et al.^26^ was employed. 200 μL of the reaction mixture (20 μL EDTA, 20 μL methionine, 20 μL NBT, 78 μL phosphate buffer, 2 μL riboflavin) and 60 μL plant sample was poured in 96 well plate. Blank was prepared by mixing all the chemicals except plant supernatant in the same quantity. Absorbance was measured at 560 nm using a micro-plate reader after seven min incubation of mixture in fluorescent light.

POD activity in shoots supernatant was determined by the method of Lagrimini^27^. Reaction mixture (40 μL guaiacol 100mM, 100 μL dH_2_O, 20 μL H_2_O_2_ 27.5 mM, and 40 μL KH_2_PO_4_ buffer 50 mM) of 200 μL was poured in 96 well plate and 20 μL of plant extract was added. Reaction mixture without extract was used as control. Absorbance was measured using a micro-plate reader at 470 nm wavelength.

### Biological profile of root and shoot

At the time of fruit collection, roots and shoots were also collected, dried, and powdered in electric blender. The sample powder was suspended in 1 mL dimethyl sulfoxide (DMSO) in eppendorf tubes and kept at room temperature. After 24 h, the mixture was centrifuged at 10,000 rpm for 10 min. The supernatant was used for the biochemical profiling.

Total phenolic content (TPC) were determined by the method of Astill et al. (2001). 20 μL of the samples, standard or blank i.e. sample, Gallic acid, or DMSO were transferred to each well of 96 well plates. In each well, 90 μL of 10 times diluted freshly prepared FC (Folin– Ciocalteu) reagent was added. The plates were incubated at room temperature for 5 min and then 90 μL of Na_2_CO_3_ solution was added to reaction mixture. The plates were then incubated for 1 h and absorbance was measured at 650 nm by using microplate reader.

Total flavonoid contents (TFC) were determined using the method as described by Almajano et al. (2008). Test sample20 μL, standard and blank were poured in the 96 well microplate and 10 μL of 10% aluminum chloride solution was added. Thereafter 10 μL of potassium acetate (1 M) solutions was added and the final volume was raised up to 200 μL by adding 160 μL of distilled water. The plate was incubated at room temperature for 30 min and optical density of samples was measured at 415 nm by microplate reader (Bioteck, USA).

The free radical scavenging activity of test sample against DPPH was determined using procedure described by Sajjad et al.*,* (2021). 10 μL of test sample, standard and blank were mixed with 190 μL of DPPH solution in the 96 well microplate. The reaction mixture was incubated in dark for 1 h at 37°C. The optical density of the samples was measured at 517 nm using microplate reader. Percent inhibition of test sample was calculated using the following formula: Where: **A=** O.D of DPPH solution with sample; **B=** O.D of negative control (containing the reagent except the sample).

$$Percent inhibition of the test sample = \left( {1 {-} A / B} \right) *10 \cdot 0$$

Total antioxidant activity (TAC) of roots and shoots of soybean and sesame plants was evaluated using protocol described by Sajjad et al. (2021). In TAC assay, 100 μL stock solution/test samples were added to Eppendorf tubes and 900 μL of TAC reagent (H_2_SO_4_, NaH_2_PO_4_.H_2_O, (NH_4_)_6_Mo_7_O_24_.4H_2_O) was then added and mixed thoroughly. The tubes were then incubated at 95 °C for 90 min. After incubation, the reaction mixtures were cooled at room temperature and then 200 μL of uniformly mixed solution were transferred to 96 well plates. The absorbance was measured at 630 nm by using micro plate reader.

Total reduction potential (TRP) of the test samples was evaluated according to the procedure described by Sajjad et al. (2023). For TRP assay, 100 μL of the test samples was mixed with 200 μL of phosphate buffer (0.2 M, pH 6.6) and 250 μL of 1% potassium ferricyanide solution. The reaction mixtures were then incubated for 20 min at 50⁰C. After incubation, the reaction mixture was acidified with 200 μL of 10% trichloroacetic acid. The resulting mixtures were centrifuged at 3000 rpm for 10 min. 150 μL of supernatant layer of each centrifuged mixture was transferred to 96 well microplates and mixed with 50 μL of 0.1% ferric chloride solution. The optical density was measured at 630 nm using microplate reader. The results were expressed as μg Ascorbic acid equivalent per mg extract (μg AAE/mg extract)

### Measurement of oil content

The 15 g seeds were oven-dried for 5 h at 50°C. The oil content was measured via non-destructive technique employing NMR analyzer (Newport 400 of Oxford Analytical Instruments) having a coil assembly of 40 mL. The seed were placed in sample holder and the oil content was recorded as percent oil present in the seeds.

### Oil extraction and Estimation of free fatty acid (FFA) contents in *E. sativa* oil

The seed were washed with distilled water and dried in oven at 70 °C for 12 h. The oil was extracted through electrical expeller. The extracted was store for biodiesel production and further analysis.

Aqueous acid-base titration was used to determine the free fatty acid content in the oil extracted from *E. sativa* seeds. Blank and sample titrations were carried out. To perform blank titration, 0.025M KOH solution (0.14 g KOH in 100 mL distilled H_2_O) was poured in a burette. Isopropyl alcohol (10 mL) was taken in a conical flask and 2-3 drops of phenolphthalein indicator (0.5g phenolphthalein in 50% ethanol) and titrated against 0.025M KOH till the solution become dusky and turned pink indicating the endpoint of the reaction. At this point, the used volume of KOH was noted. For sample titration, isopropyl alcohol and oil were taken at a ratio of 9:1 in a conical flask and 2-3 drops of phenolphthalein indicator was added. The mixture was titrated against 0.025M KOH till the end point and used volume KOH was noted. The experiments for both blank titration and sample titration were carried out thrice to find average volume of KOH used in titrations. The following equation was used to determine the free fatty acid (FFA) content of *E. sativa* crude oil^28^.

$${\rm FFA}=\left(A-B\right)\times C\div V$$

where,

A= Volume used in Sample titration, B= Volume used in Blank titration, C= Mass of KOH in g/L

V= total volume of oil used

### Biodiesel production

**Fig. 2:**
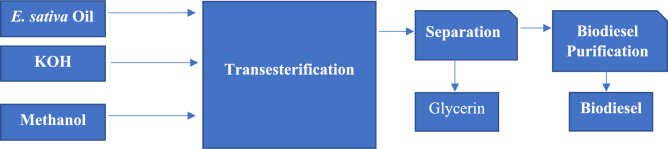
Schematic flow chart of transesterification reaction procedure.

Before transesterification reaction, 1% KOH as catalyst was mixed well with methanol. The preheated *E. sativa* oil was mixed with methanol at 6:1 and subjected to stirring for 3 h at 65˚C for transesterification reaction. The mixture of biodiesel was allowed to stand overnight to separate biodiesel from catalyst and glycerin layer. The crude biodiesel was isolated and washed with distilled water to remove impurities such as CH_3_OH, KOH, and glycerin. These impurities absorbed into water, settle down, and leave pure biodiesel at the top. This process was repeated until clear water appeared^29,30^. This procedure of biodiesel production was employed for the oils extracted from treated and non-treated *E. sativa* seeds (Figure 2). The percent biodiesel yield was calculated using the following equation.

$$\%Yield=\frac{{m}_{1}}{{m}_{2}}\times 100$$

Where, ${m}_{1}$ is the mass of biodiesel produced, and ${m}_{2}$ is the mass of oil samples used.

Fatty acid (R^1^COOH) + Alcohol (ROH) ⇋ Ester (R^1^COOR) + Water (H_2_O)

Triglyceride + R^1^OH ⇋ Diglyceride + RCOOR^1^

Diglyceride + R^1^OH ⇋ Monoglyceride + RCOOR^1^

Monoglyceride + R^1^OH ⇋ Glycerol + RCOOR^1^

Generalize equation for transesterification of triglycerides.

### Determination of fuel properties

The fuel properties of *Eruca sativa* biodiesel *viz* pour point, cloud point, flash point, viscosity, density, sulfur content, ash analysis, and acid value were determined using standard procedures of American Society for Testing and Materials (ASTM) (Table 3). These properties were compared with International Standards of Biodiesel i.e., European (EN-14214), American (ASTM D-6751), and Chinese GB/T 20,828 standards.

**Table 3:** Procedures adopted for the determination of fuel properties.

**S. No.**	**Biodiesel property**	**Procedure**
1	Pour point	ASTM D-97
2	Cloud point	ASTM D-2500
3	Flash point	ASTM D-93
4	Viscosity	ASTM D-445
5	Density at 40°C	ASTM D-5002
6	Sulfur content	ASTM D-4294
7	Ash analysis	ASTM D-482-07
8	Acid value	ASTM D D-974

For the determination of pour point, the biodiesel was kept at room temperature for 24 h. After that, the biodiesel was poured into a test jar until its level reached a marked line at 5.5 cm above the bottom. The jar was sealed with cork and a thermometer was inserted in such a manner that its bulb remains 3 mm below the marked line, and the temperature was recorded. The jar was kept in jacket, preventing contact between the jacket and jar through a gasket. For observation, the jar was held horizontally for 5 s. The observation of flowing ability of biodiesel was initiated at a temperature at least 9 °C above the anticipated pour point. The biodiesel was allowed to cool until it completely ceased to flow, determining the pour point.

For recording cloud point, 10 cm^3^ biodiesel was taken in 100 cm^3^ beaker and kept in deep freezer. The biodiesel sample was examined at intervals and the temperature was recorded when the bottom of the beaker became hazy.

Flash point of the biodiesel was measured using automated Pensky Martens closed cup apparatus, for which the temperature range was set between 60-190 °C. The biodiesel sample was filled in a brass test cup and the lid was closed. The sample was heated and stirred. The ignition source was introduced at regular interval into the test cup with concurrent interruption of stirring, till the flash is detected, which spread throughout the cup. The temperature was recorded at the appearance of flash.

Redwood viscometer was used for the determination of viscosity of biodiesel. Viscosity value is the time needed for 50 ml biodiesel to come out of viscometer at standard temperature. The biodiesel was poured in to viscometer cup, heated electrically, and the pressure was kept constant. The time for the flow of 50 ml biodiesel was recoded at 40 °C.

The density of the biodiesel sample was determined through digital density analyzer (PAAR, DMA 38). The biodiesel sample (0.7 mL) was poured in to an oscillating sample tube and the density was measured by change in the mass of oscillating tube which altered the oscillating frequency.

The amount of sulfur in biodiesel was estimated by energy-dispersive X-ray fluorescence spectrometry (EDXRF) (OXFORD, Model Lab-X3000). The biodiesel sample was placed in X-rays beam and the resultant excited rays were measured followed by comparison with X-rays count obtained from standards in the concentration ranges of 0.015-0.1 % and 0.1-5.0 %.

For the determination of ash content, 10 g biodiesel sample was ignited at 800 °C in dry and pre-weighed silica crucible until only ash was left. The crucible was allowed in a desiccator at room temperature and weighed. The difference between the weight of crucible before and after ignition was used to calculate the ash percentage of the original biodiesel mass.

The acid value was estimated by dissolving 10 g of biodiesel in a 30 cm^3^ mixture of isopropanol and toluene containing small amount of water. This solution was titrated against KOH to the end point as indicated by pink color due to phenolphthalein indicator.

### Characterization of biodiesel

FT-IR spectra of *E. sativa* oil and biodiesel were attained on Burker-Platinum-ATR spectrometer at a scanning rate of 15 at 1cm^−1^ to analyze their functional groups and structural arrangement. A drop of oil and biodiesel was placed directly on spectrometer and the spectra were recorded in a wavenumber range of 4000-500 cm^−1^.

The fatty acid methyl esters (FAME) profile of biodiesel was determined by GC-MS through QP2010 Plus spectrometer fitted with capillary column of 0.2 µm thickness, length of 30 m, and, inner diameter of 0.2 µm. The FAMES were identified by a search of NIST17R.lib and NIST17M2 standard mass spectrum database. High purity (99.99%) helium gas was used as carrier at a flow rate of 1.5 mL/min. The injection temperature was set as 250 °C, whereas interface temperature was kept as 280°C. The oven temperature was kept at 50 °C with a hold time of 5 min and the scan range was m/z 40-800.The biodiesel sample (1 µL) with a split ratio of 20:1was injected and the spectra were obtained after complete scanning of biodiesel.

^1^H and ^13^C NMR spectra of *E. sativa* biodiesel were recorded at 298 K on Bruker-Avance 300 MHz spectrometer. Tetramethylsilane (TMS) and Deuterated chloroform (CDCl_3_) were used as internal standard and solvent, respectively. After vertexing with CDCl_3_, the biodiesel was transferred to NMR sample tube to record NMR spectra. ^1^H (300MHz) spectra were recorded at a flip angle of 30°, recycle delay of 1 s, scan number of 10, and pulse duration of 30°. ^13^C NMR (75MHz) spectra were recorded at a flip angle of 30°, recycle delay of 1.89 s, scan number of 10, and pulse duration of 30°.

### Statistical analysis

All experiments were carried out in triplicate. The field experiment was performed in a Randomized Complete Block Design (RCBD) to evaluate the effect of CuS NPs and CuSO_4_ on the agronomic parameters and oil content of *Eruca sativa*. Analysis of variance (ANOVA) was used to determine significant difference between treatments. To compare treatment, the results were further analyzed statistically through HSD test at *P*≤ *0.05* using STATISTIX 8.1® (Hill and Lewicki 2006).

## Results and discussion

### Characterization of CuS NPs

**Fig. 3:**
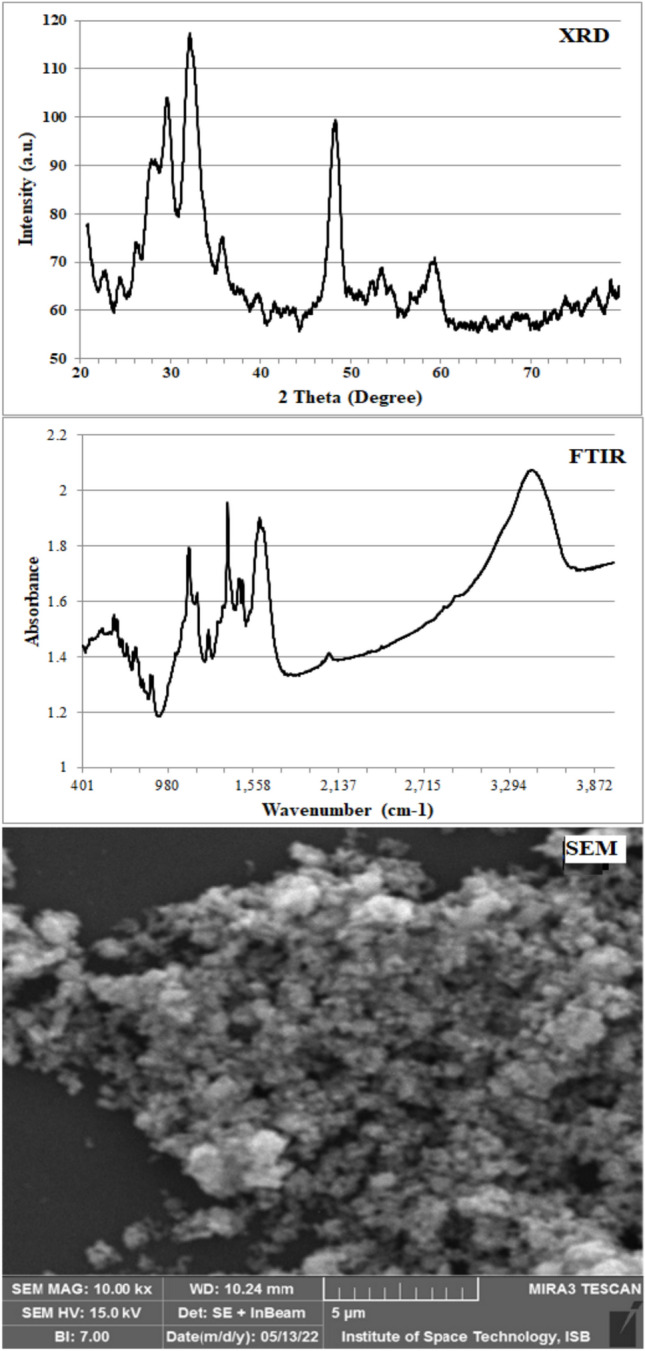
Characterization analysis of CuS NPs by XRD diffractogram, Fourier transform infrared spectroscopy (FTIR) spectrum, and scanning electron microscopy (SEM) image.

SEM images present roughly spherical forms of CuS NPs that were moderately aggregated (Figure 3). Chemical approaches for synthesis of NPs present good morphological products, less aggregated, and smaller size. Energy dispersive X-ray (EDX) analysis percents composition of Cu and S elements in CuS NPs. The surface showed 48.51% Cu and 39.56% S by weight. The precent composition reflects atomic arrangement and purity of NPs^31^.

The XRD analysis of CuS NPs revealed diffraction peaks at 27*.*68º, 29.23º, 31.78º, 47.73º, 52.67º, and 59.21º. These diffraction peaks align with lattice planes (101), (102), (103), (107), (108), and (116), respectively (Figure 3). JCPDS card 06-0464 support the diffractogram and corresponds to hexagonal crystal lattice of CUS NPs (^32–34^. The average size calculated by equation determines 4.32 nm NPs**.**

FTIR peaks presented broad band in the region of 3450 cm^−1^ corresponds to the stretching vibrations of the OH group and can be attributable to adsorbed water or surface-bound hydroxyl ions. The bands located at 1600 cm^−1^ and 1384 cm^−1^ represent the O–H bending mode that indicates Cu-O-H structure (Figure 3). A band at 1124 cm^−1^ suggests asymmetric stretching vibration of SO_4_^-^ species whereas the band at 617 cm^−1^ can be attributed to Cu-S stretching vibrations^34,35^.

### Growth and yield attributes of *Eruca sativa*

Physical growth and yield parameters of *E. sativa* exposed to CuS NPs and CuSO_4_ under field environment are shown in Table 4. The application of CuS NPs markedly increased the growth and yield parameters of *E. sativa*. CuS NPs improved the root length (20.23%), shoot length (21.17%), branches per plant (45.64%), and dry weight per plant (49.46%) as compared to untreated control plants. Similarly, yield parameters such as siliqua per plant (48.68%), seeds per siliqua (18.16%), and oil percentage (18.27%) also increased in response to CuS NPs with respect to control plants. Although, the application of CuSO_4_ also improved in the aforementioned parameters compared to control, yet lower than CuS NPs treated plants. Cu is an essential micronutrient for plant and acts as cofactor for different enzymes, photosynthetic machinery, and cytochrome oxidase of the respiratory electron transport chain. Plants produce reactive oxygen species (ROS) in response to Cu stress^36,37^. Rameen et al.^15^ reported increased growth and yield parameter in *Brassica napus* in response Cu NPs at below threshold concentration. Cu NPs increased the number pod per plant in *Glycine max* upon exposure to soil applied Cu NPs^38^. However, some studies have shown that Cu NPs reduced root and shoot length, water content, and biomass in *Lactuca sativa* and wheat^39–41^. The S component of CuS NPs mitigated the Cu toxicity in the current study as various studies revealed that sulfur application alleviates heavy meat toxicity^42–44^. The exact mechanism through which Cu NPs improve agronomic parameters is still to be uncovered. However, it is assumed that Cu interferes with plant biochemical processes and enzymes that are involved in biochemical processes including lipid production. Sulfur plays important role in the synthesis of oil, protein, vitamins, and aromatic compounds in plants. It is also a component of three important amino acids,cystine, cysteine, and methionine. Enhanced oil content in *E. sativa* seed may also be attributed to the role of S in oil biosynthesis.

**Table 4:** Effect of CuSO_4_ and CuS NPs on the growth and agronomic parameters *Eruca sativa*. The similar letters within the column mean no statistical difference between treatments in the LSD test (p ≤0.05).

	**Growth parameters**				**Yield attributes**		
	**Root length (cm)**	**Shoot length (cm)**	**branches (Plant**^**−1**^**)**	**Dry weight (g Plant**^**−1**^**)**	**Siliqua (Plant**^**−1**^**)**	**Seeds (Siliqua **^**−1**^**)**	**Oil %**
**Control**	16.8±0.84b	128.2±5.12c	3.33±0.01c	13.93±0.27b	128.46±5.13c	18.33±0.91b	34.59±1.38b
**CuSO**_**4**_	17.4±0.52b	138.06±4.14b	3.93±0.15b	14.06±0.42b	135.33±6.76b	19.1±0.57b	34.73±1.73b
**CuS NPs**	20.2±1.01a	155.35±7.76a	4.85±0.14a	20.82±0.83a	191±5.73a	21.66±0.86a	40.91±1.73a

### Elemental analysis

The roots of *E. sativa* grown in presence of CuS NPs accumulated highest Cu content (54.46±1.63 mg/kg) followed by shoot (34.53±1.72 mg/kg) and seeds (9.4±0.47 mg/kg). Cu accumulation was observed in the same manner in *E. sativa* treated with CuSO_4_ (Table 5). As the roots are in direct contact with soil seems to accumulate more amount of Cu whereas seed being farthest from root and develop later in the life tends to accumulate the least amount of Cu. Moreover, dissolution of metals from nanoparticles also promotes uptake of Cu by roots^45^. The less accumulation of Cu in salt treated plants as compared to NPs treated plants points that the Cu ions released from salt were less available either due to leaching or binding with other molecules while Cu slowly dissolute from NPs conforming Cu availability throughout the life. These findings are aligned with Ogunkunle et al.^46^, where they found higher Cu concentration in the root followed by leaves and seed of cowpea upon exposure to Cu NPs.

**Table 5:** Cu (mg/kg) in soil, root, shoots, and seed of *Eruca sativa* exposed to CuS NPs and CuSO_4_. Bioaccumulation and translocation factors of Cu in *Eruca sativa*. The similar letters within the row mean no statistical difference between treatments in the LSD test (p ≤0.05).

	**Soil**	**Root**	**Shoot**	**Seed**	**BAF**	**TF**
**Control**	1.41±0.02d	33.66±1.34a	16.42±0.49b	2.93±0.14c	37.59	0.57
**CuSO**_**4**_	3.83±0.11d	42.2±2.11a	23.06±0.92b	4.76±0.19c	18.28	0.65
**CuS NPs**	5.63±0.22d	54.46±1.63a	34.53±1.72b	9.4±0.47c	17.47	0.80

Bioaccumulation factors (BF) ranged from 17.47 to 37.59, whereas translocation factors (TF) ranged from 0.57 to 0.8 (Table 5). TF was determined to assess the capability of *E. Sativa* for Cu translocation from root to shoot. It is evident from the results that highest TF was observed in CuS NPs treated plants suggesting high translocation efficiency of *E. sativa*. Consequently, it is concluded that translocation capability of *E. sativa* increased with increase in the amount of Cu in the soil. Nanoparticles accumulate in roots and translocated to the shoot and seeds^47,48^. NPs type, concentration, size surface charge, ion dissolution efficiency as well as plant species affect the absorption, bioaccumulation, and translocation of metals in plants^49–52^. Larger NPs struggle to pass through tiny pores in the cell wall^53^ whereas smaller size NPs have better potential to transport^54^. Nanoparticles adhere to root surface, enter the cell wall and move between cell wall through plasmodesmata and transported shoot via xylem^53^. Wang et al.^55^ reported the transport of Cu NPs from root to shoot through xylem.

### Biochemical analysis of Plants

The protein content in *E. sativa* treated with CuS NPs increased (175.16±8.5 mg/g FW) as compare to untreated control plants (63.88±3.19 mg/g FW). Similar results were noted for enzymatic antioxidants, SOD and POD in response to externally applied CuS NPs (Table 6). Moreover, non-enzymatic antioxidants, phenolics and flavonoids also increased in the root and shoot of *E. sativa* upon exposure to CuS NPs. The root of *E. sativa* showed an increase of TPC 44.26% while shoots depicted 15.33% increase of TPC as compared to control plants. Similarly, TFC increased by 11.62% and 8.04% in roots and shoots, respectively as contrast to control plants. The root and shoot of *E. sativa* also showed increase of DPPH free radical scavenging activity, TAC and TRP upon exposure to CuS NPs (Table 7). A slight increase in the above parameters was also observed in response to CuSO_4_ treatment. However, this increase was negligible when compared with CuS NPs treatments.

**Table 6:** Effect of soil applied CuS NPs on the Protein content, SOD, POD, Total Phenolic and Total Flavonoid Contents of *E. sativa* under field condition. The similar letters within the column mean no statistical difference between treatments in the LSD test (p ≤0.05).

**Treatment**	**Protein Contents (mg/g FW)**	**SOD (nM/min/gFW)**	**POD (nM/min/g FW)**	**Total Phenolic Contents** **(μg QE/mg DW)**		**Total Flavonoid Contents (μg GAE/mg DW)**	
				**Root**	**Shoot**	**Root**	**Shoot**
**Control**	63.88±3.19c	0.022±0.001c	0.52±0.026c	97.46±4.87c	164.35±4.93c	28.9±1.15b	122.36±6.11b
**CuSO**_**4**_	94.58±4.72b	0.05±0.002b	0.58±0.017b	125.2±3.75b	174.97±6.99b	28.96±0.86b	122.91±3.68b
**CuS NPs**	175.16±8.5a	0.63±0.031a	0.74±0.037a	140.6±5.62a	186.8±9.34a	32.26±1.61a	132.14±5.28a

**Table 7:** Effect of soil applied CuSO_4_ and CuS NPs on DPPH radical scavenging, TAC, and TRP of *Eruca sativa* under field condition. The similar letters within the column mean no statistical difference between treatments in the LSD test (p ≤0.05).

**Treatment**	**DPPH** **(% inhibition)**		**TAC** **(µg AAE/mg DW)**		**TRP** **(µg AAE/mg DW)**	
	**Root**	**Shoot**	**Root**	**Shoot**	**Root**	**Shoot**
**Control**	25.01±1.25c	36.19±1.8b	307.18±12.28c	397.05±11.91b	173.58±8.67c	222.84±8.91c
**CuSO**_**4**_	30.91±1.23b	39.87±1.19b	383.26±12.49ab	406.53±16.26b	186.96±5.6b	262.49±13.12b
**CuS NPs**	36.93±1.1a	45.87±1.83a	407.26±16.29a	455.06±18.2a	208.29±8.33a	287.88±8.63a

Faizan & Hayat^56^reported that metallic NPs enhance protein content in tomato by interfering in transcription and translation processes. In another study, Salama^57^, found that Ag NPs augmented protein content in common bean and corn up 60 ppm and decreased above this dose. A concentration dependent increase in the amount of protein was also observed by Leopold et al.^58^ in soybean upon exposure to metallic nanoparticles. At cellular level, SOD and POD provide the first defense mechanism against oxidative damage^59^. SOD catalyzes the dismutation of superoxide radicals O_2_^**.**^ to molecular oxygen and hydrogen peroxide. POD being a key enzyme in antioxidant enzyme convert hydrogen peroxide to H_2_O and O_2_. In the current study there was a clear increase in the SOD and POD activities of the plant treated with CuS NPs. Similar results have been reported by Kim et al^60^ in *Cucumis sativus.* A significant increase in SOD gene expression was also observed by Nair and Chung^61^ in pea upon treatment with different concentration of Cu NPs. TPC and TFC are non-enzymatic antioxidants which protect the plants against stresses. They affect plant growth and development through the life^62,63^. The amount of TPC and TFC augmented in Brassica^64,65^, *Abelmoschus esculentus*^66^*, Ocimum basilicum* (Nazir et al. 2012), cucumber Zhao et al.^67^^,^ and rice^68^ upon exposure to different metallic NPs including Cu NPs. The variation in the amount of TPC and TFC in response to NPs is possibly due to ROS production. Still, this variation depends on the type of NPs, mode of application, and age and specie of the plant. Upon entry in to the cell, these NPs cause oxidative stress by interacting with cell organelles^69^. The plant defense system gets activated and produces antioxidant such as phenolics and flavonoids under stress^70^. In the current study, the increase in DPPH free radical scavenging activity, TAC, and TRP under the elicitation of CuS NPs shows exertion of oxidative stress that do not negative effect plant growth and yield as depicted by agronomic parameters. ROS produced in response to stress may also act as signaling molecules^71^. The increase in antioxidant activities indicate strong detoxification mechanism that protect the plant cells from eternally applied Cu stress.

### Biodiesel synthesis and yield

The oil obtained from *E*. *sativa* plants seeds were converted to biodiesel. Prior to biodiesel synthesis, the free fatty acid (FFA) was determined and found to be 0.31 mg KOH/g. The quality and yield of biodiesel directly depends on amount of FFA which affect the rate of biodiesel synthesis. The FAA value up to 3% is considered appropriate for efficient conversion of oil to biodiesel in a single step transesterification reaction. The FFA content above this value leads to saponification which makes the separation difficult^72,73^. Owon et al.^74^ reported 1.13% free fatty acids content in rocket seed oil. Our value of FFA content is within the safe limit for single step transesterification reaction. The percent yield of biodiesel was 85.16, 85.31 and 84.98 for the oil extracted from control, CuSO_4_, and CuS NPs treated *E. sativa* plants, respectively. Tariq et al.^75^ also observed 83.3% yield in a study using rocket seed oil as feedstock for biodiesel production.

### Fuel properties

The fuel properties of *E. sativa* oil biodiesel are presented in table 6 and compared with international standard i.e., American (ASTM- 6751), European (EN- 14214), China (GB-T) and published studies. The determination of fuel properties is considered essential before applications in diesel-based engines.

The lowest temperature at which a fuel is able to flow is pour point while the temperature at which crystals formation starts to form precipitate is known as cloud point. The pour points were found to be between −4.01°C to −4.32°C, while cloud points were from 2.93 ^o^C to 3.3 ^o^C (Table 8). These values are within the recommended limits for standard biodiesel. For low temperature operation of fuel, pour point and cloud points are commonly used^76^. The parameters show the least temperature at which a fuel ignites efficiently. The low pour and cloud points in this study indicate that *E. sativa* biodiesel can be used in a moderate cold climate tropical country like Pakistan.

**Table 8:** Fuel properties of *E. sativa* biodiesel synthesized from oil extracted from the seeds of control, CuSO_4_, and CuS NPs treated plant and its comparison with international standard biodiesel.

**Property**	**E. sativa biodiesel (control)**	**E. sativa biodiesel (CuSO**_**4**_ **)**	**E. sativa biodiesel (CuS NPs)**	**American (ASTM D-6751)**	**European EN- 14214**	**China GB/T 20,828-2007**
Pour point (^o^C)	−4.01	−4.32	−4.11	−15 to 16	-	-
Cloud point (^o^C)	2.93	3.3	3.01	−3 to 12	-	-
Flash point (^o^C)	133	132	130	100–170	≥ 120	≥ 130
Viscosity (at 40 °C mm^2^ s^−1^)	5.33	5.22	5.41	1.9–6.0	3.4–5.0	-
Density (at 40°C g/m^3^)	0.87	0.86	0.86	0.86–0.90	0.86–0.89	-
Sulfur content (wt. %)	0.017	0.11	0.013	≤0.05	0.020	0.020
Ash analysis (mass %)	0.01	0.012	0.014	0.02	0.02	-
Acid value (mg KOH g^−1^)	0.14	0.19	0.17	≤0.5	≤0.5	≤ 0.8

Flash point is the temperature at which the fuel vaporizes and is a significant parameter in safety, handling, and storage of fuel and inflammable substances. The flash points of *E. sativa* biodiesel obtained from seeds of plants of different treatments are close to each other and are well in the range of international standards (Table 6). Viscosity is another important feature of fuel as both low and higher viscosity affect the proper operation of engine. It influences the atomization of biofuel after injection to diesel engine. High viscosity results in incomplete combustion due to poor oxygen supply which lead to deposition in the diesel engines (Knothe and Steidley, 2005) while low viscosity causes low combustion due to low penetration which lead to black smoke emission. In this study, the viscosity of biodiesel obtained from seeds of plants treated with CuS NPs was little bit higher than control (Table 8). These values are also in the range of international standards for biodiesels.

Density of biodiesel affects the atomization of fuel during combustion and engine performance^77^. The densities of biodiesel of *E. sativa* seed oil were found same and comparable to international standard of biodiesel (0.86–0.90 g/m^3^) (Table 8). The sulfur content is present in biodiesel primarily due to the existence of phospholipids in oil^78^. The sulfur content was observed 0.017 for control,0.011 for CuSO_4_; and 0.013 wt.% for CuS NPs treated *E. sativa* oils that also lies within the ideal range of international standards. Ash content indicates the presence of inorganic contaminants in biodiesel. During combustion process, these inorganic compounds oxidize quickly and lead to ash formation. The ash accumulates in engine that reduces efficiency of engine^79,80^. The results in table 6 show that ash content of *E. sativa* biodiesel lies within the recommended international standard range.

The acid value represents the quantity of free fatty acid in biodiesel. High acid number (≥ 0.50 mg KOH g−1) may negatively affect engine performance due to corrosion. The acid value in current study were observed to be in the range of 0.14-0.19 mg KOH g^−1^, which are comparable to international standard for biodiesel (Table 8).

Similar result for fuel properties of biodiesel synthesized from *Eruca sativa* seed oil biodiesel is reported by^81,82^, and Tariq et al.^75^.

### Characterization of biodeisel

#### FT-IR analysis of Eruca sativa oil and biodeisel

**Fig. 4:**
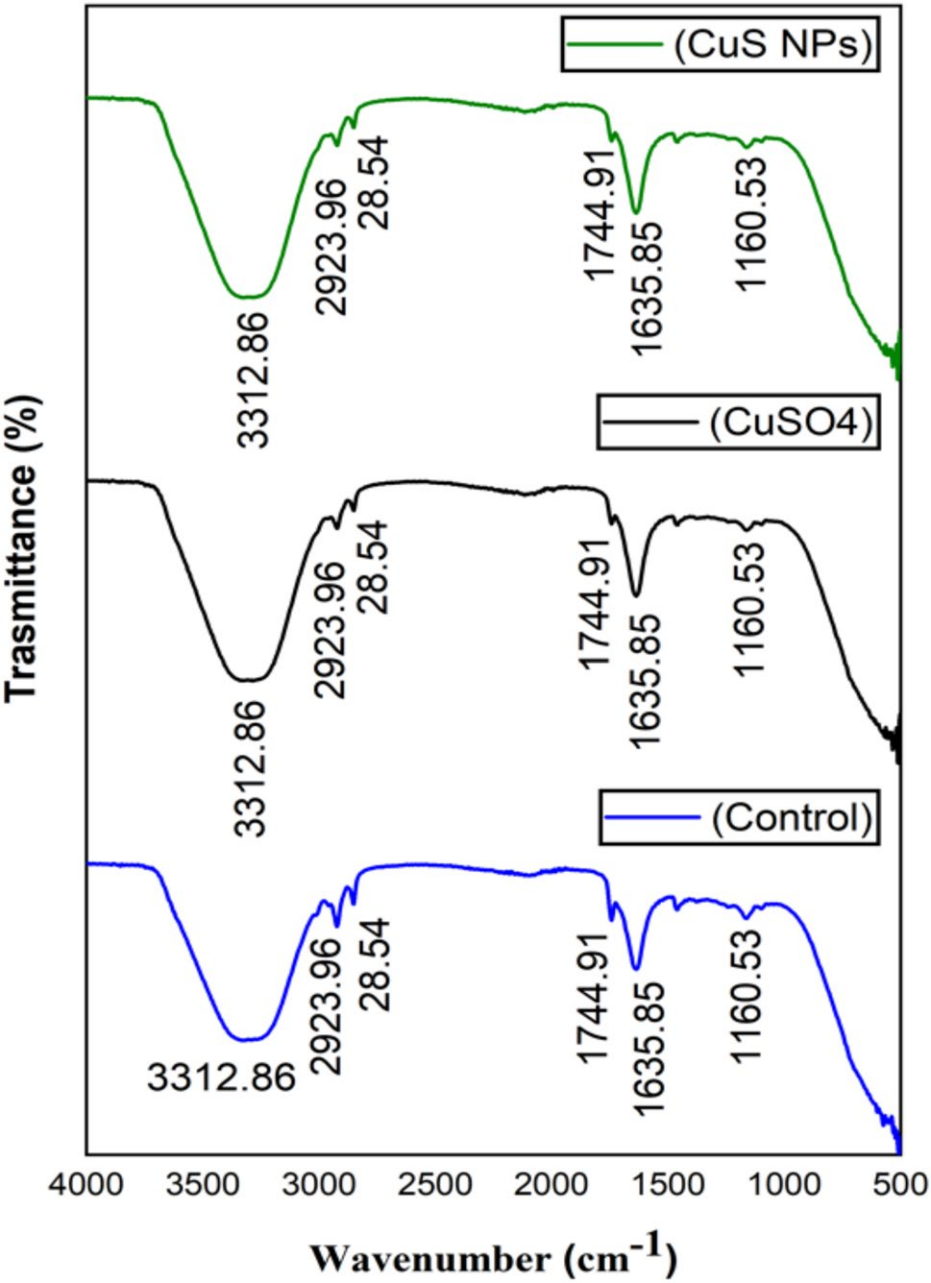
FT-IR spectra *E. sativa* oil extracted from control, CuSO_4_ and CuS NPs treated plants.

**Fig. 5:**
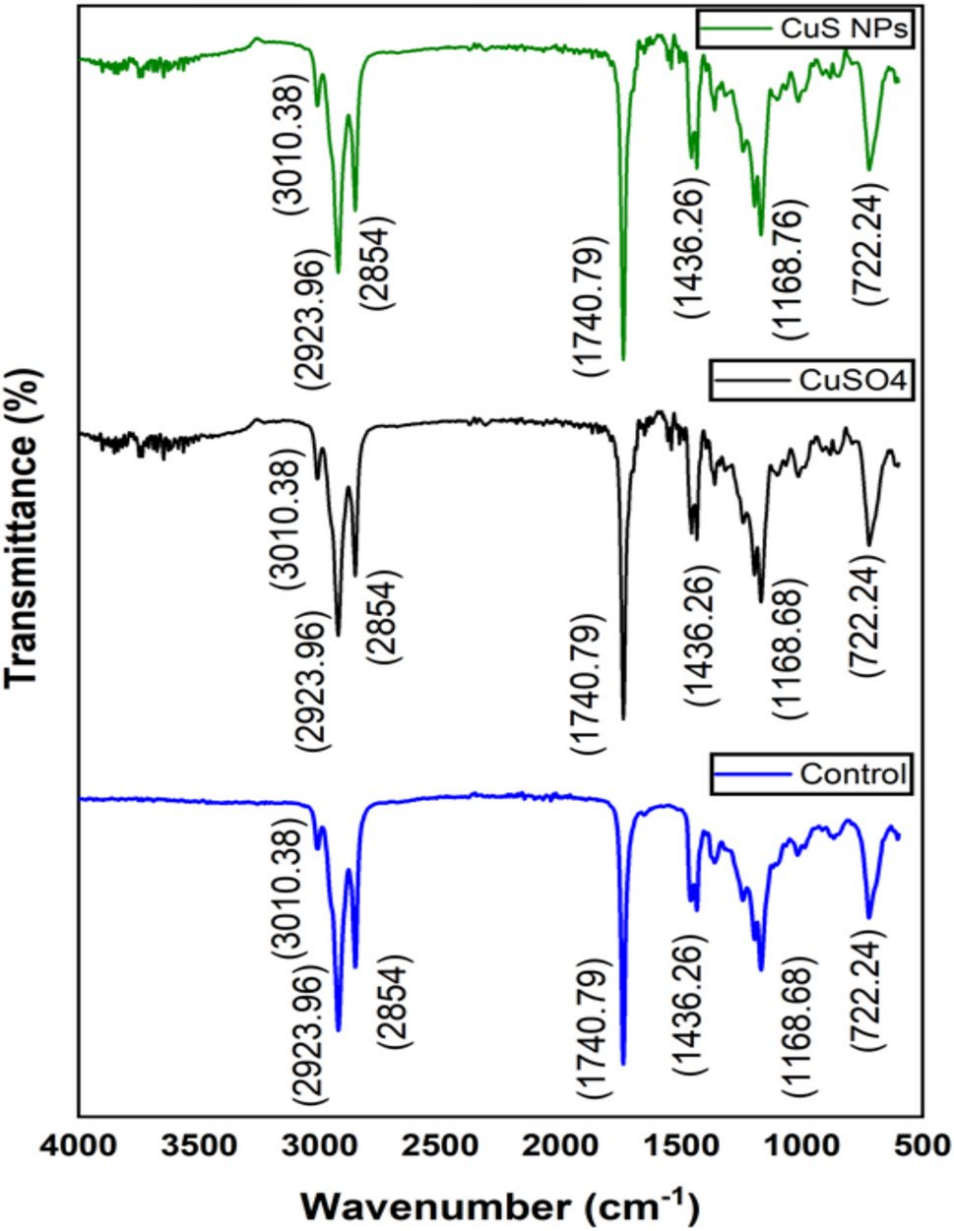
FT-IR spectra *E. sativa* biodiesel prepared from oil extracted from control, CuSO_4_ and CuS NPs treated plants.

FT-IR analysis was used to identify the functional groups and bands attributed to different bending vibrations and stretching in the oil and biodiesel. Both *E. sativa* oil and biodiesel showed nearly similar FT-IR spectra, yet differences were observed in both spectra (Figure 4 and 5). The peaks in oil at 3312.86 cm^−1^ and 1635.85 cm^−1^ corresponding to O-H stretching vibration and C=C stretching respectively, disappeared in biodiesel, and the emergence of new peaks at 3010.38 cm^−1^ (stretching vibration of CH), 1426.32 cm^−1^ and 722.24 cm^−1^ (bending vibrations of CH_2_) is indicative of oil conversion to biodiesel^83^. The absorption band of oil in the regions of 1744.91 cm^−1^ and 1160.53 cm^−1^ shifted to 1740.79 cm^−1^ and 1168.76 cm^−1^ in biodiesel (Figure 4 and 5). Similar result of peaks disappearance in the oil spectra, emergence of new spectra in biodiesel, and shifting of peaks in *E. sativa* oil and biodiesel were observed by^81^ and Tariq et al.^75^. The biodiesel FT-IR spectra confirm successful conversion of *E. sativa* oil to biodiesel. It is concluded that CuSO_4_ and CuS NPs treatment did not affect chemical structure of oil and biodiesel, and its performance as fuel.

#### GC-MS analysis of biodiesel

**Fig. 6:**
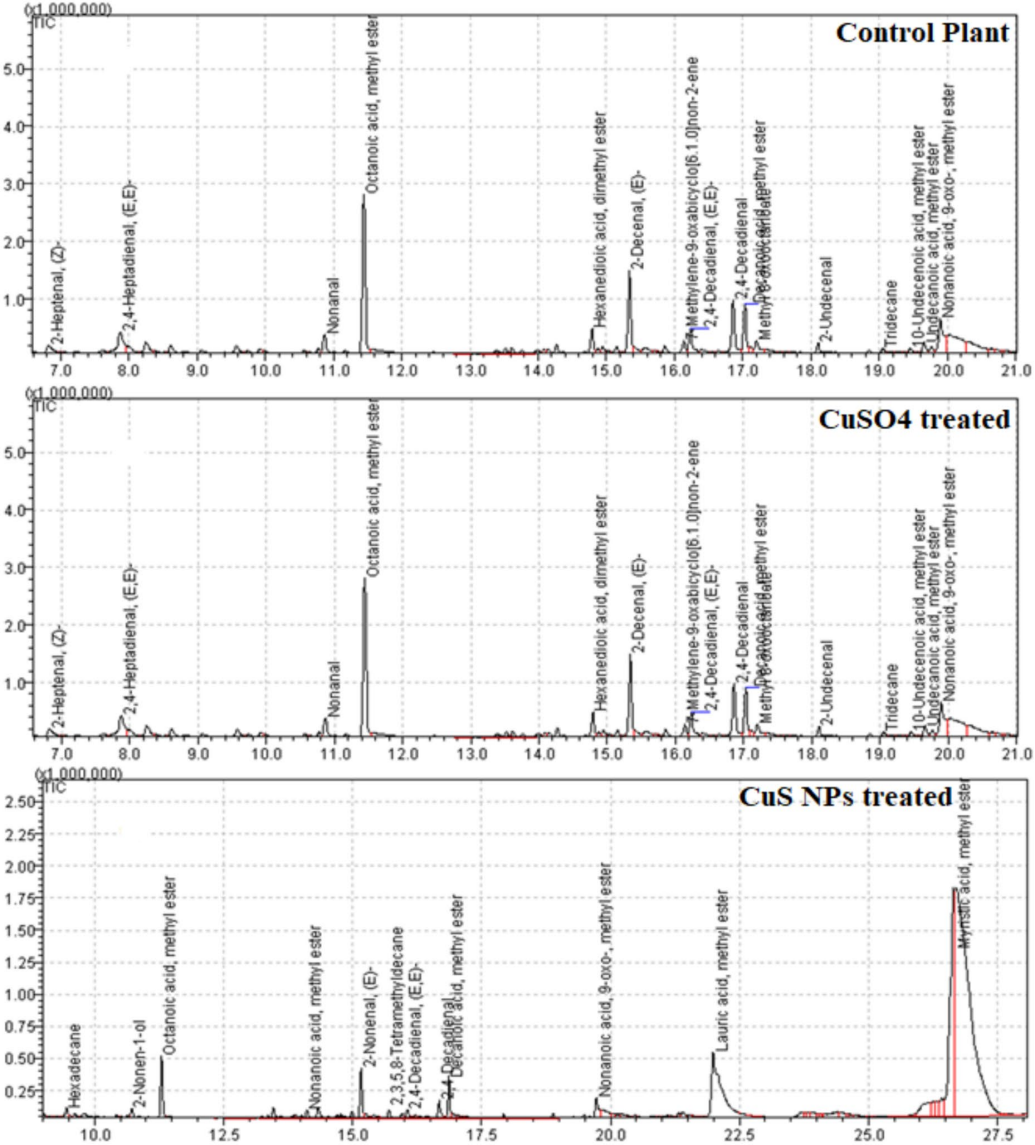
Chromatogram of *E. sativa* biodiesel prepared from oil extracted from control plant; CuSO_4_ treated plants; and CuS NPs treated plant seeds.

The fatty acid methyl ester (FAMEs) composition of *E. sativa* biodiesel was determined through GC-MS. The GC-MS analyses showed that the biodiesel composition *E. sativa* oil is not affected markedly by CuS NPs and CuSO_4_ treatments. The results indicate that FAMEs remain consistent across all treatments indicating that primary fatty acid profile remains intact despite the salt or NPs treatment. However, biodiesel obtained from oil extracted from *E. sativa* seeds grown with CuS NPs lead to variations in the relative abundances (% concentration) of specific compounds (Table 9; Figure 6). GC-MS identified up to sixteen compounds in *E. sativa* biodiesel prepared from the oil extracted from control and treated plants. Some of the compounds showed slight variation while other indicated marked variation in percent concentration. For instance, the concentration of Octanoic acid, methyl ester was 43% in control oil and 1.13% in *E. sativa* oil treated with CuS NPs. Similarly, the percent concentrations of Nonanoic acid, 9-oxo-, methyl ester, 2,4-Decadienal, (E, E)-, and 2,4-Decadienal were 3.74%, 5.37%, and 11.25%, respectively, in control oil, while 0.18%, 0.16%, and 0.34% respectively, in oil of CuS NPs treated plants (Table 9). Moreover, Lauric acid, methyl ester (16.06%) was present in the biodiesel synthesized from the oil extracted from *E. sativa* seeds oil treated with CuS NPs while absent in control and CuSO_4_ treated plant oil. CuS NPs treatment revealed a more pronounced impact on improving unsaturated FAMEs, which may enhance the quality of biodiesel in the term of oxidative stability and cold flow property^84^. Meher et al.^85^ reviewed that transesterification reaction for biodiesel production is affected by molar ratio of oil to alcohol, alcohol type, reaction time and condition, reactants purity, catalyst, and temperature.

**Table 9:** GC-MS Fatty acid profiles of biodiesel synthesized from oil extracted from control, CuS NPs and CuSO_4_ treated *E. sativa* plant seeds.

	ID#	Name	R. Time	Area	Conc. (%)	m/z
Control	1	2-Heptanal, (Z)-	6.82	119041	1.83	41.00
	2	2, 4-Heptadienal, (E, E)-	7.87	461330	7.11	81.00
	3	Nonanal	10.86	152050	2.34	57.00
	4	Octanoic acid, methyl ester	11.44	2840265	43.78	74.00
	5	Hexanedioic acid, dimethyl ester	14.79	169209	2.61	59.00
	6	2-Decenal, (Z)-	15.34	448753	6.92	55.00
	7	7-Methylene-9-oxabicyclo [6.1.0] non-2-ene	16.13	62580	0.96	79.00
	8	2,4-Decadienal, (E, E)-	16.23	348655	5.37	81.00
	9	2,4-Decadienal	16.85	729766	11.25	81.00
	10	Decanoic acid, methyl ester	17.03	595490	9.18	74.00
	11	Methyl 8-oxooctanoate	17.2	101307	1.56	74.00
	12	2-Undecenal	18.1	44306	0.68	57.00
	13	Tridecane	19.05	39699	0.61	57.00
	14	10-Undecanoic acid, methyl ester	19.45	21374	0.33	55.00
	15	Undecanoic acid, methyl ester	19.66	111138	1.71	74.00
	16	Nonanoic acid, 9-oxo-, methyl ester	19.89	242595	3.74	74.00
CuSO4	1	2-Heptanal, (Z)-	6.11	119132	1.79	41.00
	2	2, 4-Heptadienal, (E, E)-	7.66	461241	7.13	81.00
	3	Nonanal	11.05	152113	2.28	57.00
	4	Octanoic acid, methyl ester	10.95	2841343	43.80	74.00
	5	Hexanedioic acid, dimethyl ester	14.5	169198	2.59	59.00
	6	2-Decenal, (Z)-	16.34	448667	6.93	55.00
	7	7-Methylene-9-oxabicyclo [6.1.0] non-2-ene	16.22	62470	0.95	79.00
	8	2,4-Decadienal, (E, E)-	15.23	348722	5.40	81.00
	9	2,4-Decadienal	17.77	729842	11.29	81.00
	10	Decanoic acid, methyl ester	17.1	595480	0.2	74.00
	11	Methyl 8-oxooctanoate	16.11	101298	1.60	74.00
	12	2-Undecenal	17.1	44311	0.71	57.00
	13	Tridecane	18.32	39687	0.62	57.00
	14	10-Undecanoic acid, methyl ester	18.11	21402	0.33	55.00
	15	Undecanoic acid, methyl ester	20.54	111190	1.71	74.00
	16	Nonanoic acid, 9-oxo-, methyl ester	18.73	242487	3.64	74.00
CuS NPs	1	Hexadecane	9.45	38684	0.11	43.00
	2	2-Nonen-1-ol	10.72	29796	0.09	57.00
	3	Octanoic acid, methyl ester	11.29	382704	1.13	74.00
	4	Nonanoic acid, methyl ester	14.12	43656	0.13	74.00
	5	2-Nonenal, (E)-	15.16	110986	0.33	55.00
	6	2,3,5,8-Tetramethyldecane	15.7	25497	0.08	57.00
	7	2,4-Decadienal, (E, E)-	16.06	55507	0.16	81.00
	8	2,4-Decadienal	16.68	113339	0.34	81.00
	9	Decanoic acid, methyl ester	16.86	213595	0.63	74.00
	10	Nonanoic acid, 9-oxo-, methyl ester	19.72	62337	0.18	74.00
	11	Methyl tetradecanoate	26,72	2132517	6.32	74.00
	12	Pentadecanoic acid, methyl ester	28.95	1006599	2.99	74.00
	13	Lauric acid, methyl ester	21.99	5415035	16.06	81.00
	14	Myristic acid, Methyl ester	26.69	24088935	71.44	81.00

#### NMR analysis

**Figure 7:**
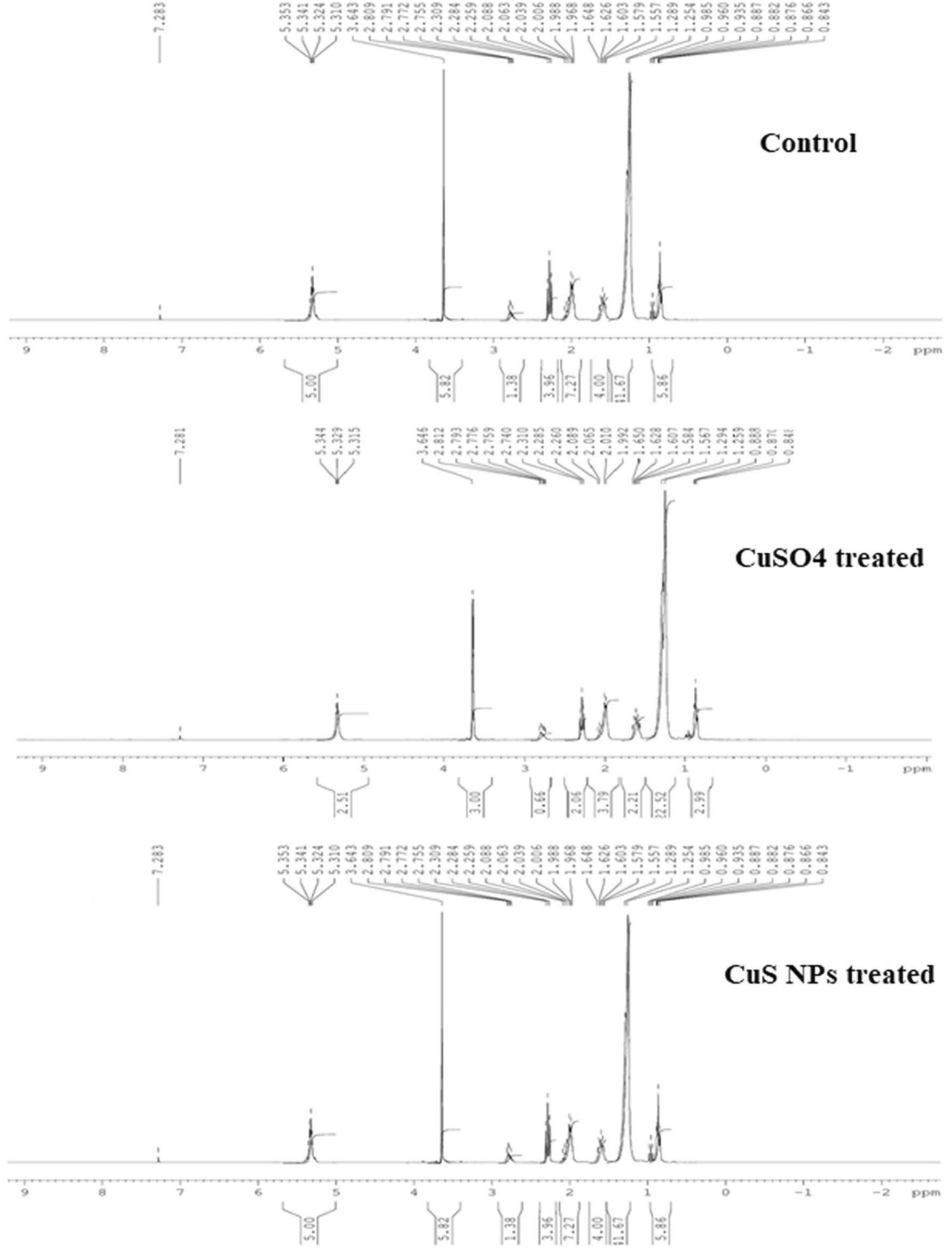
^1^H NMR spectrum of E. sativa oil biodiesel prepared from oil extracted from control, CuSO_4_, and CuS NPs treated plants seeds.

**Fig. 8:**
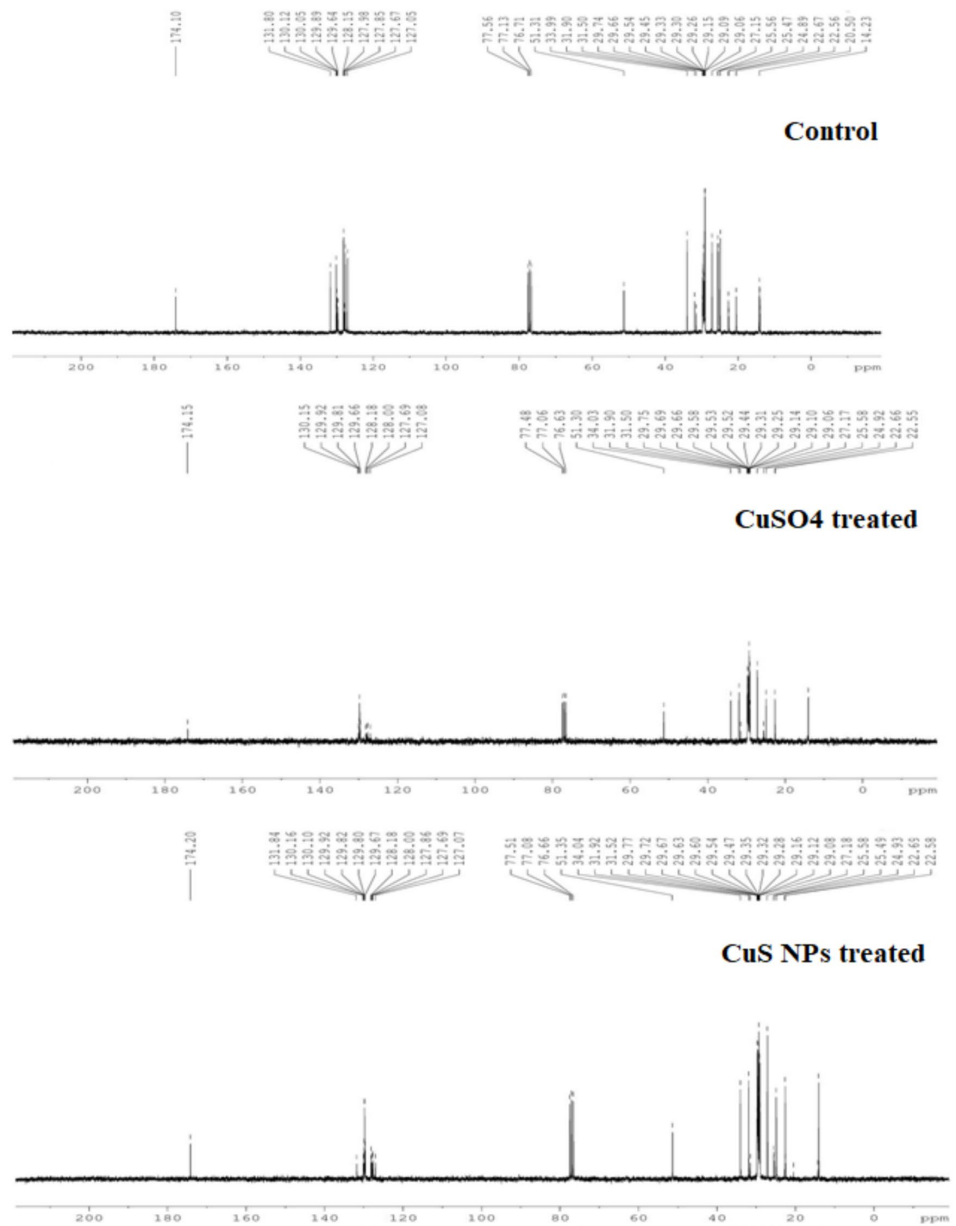
^13^C NMR spectrum of *E. sativa* oil biodiesel prepared from oil extracted from control, CuSO_4_, and CuS NPs treated plants seeds.

The biodiesel was characterized by ^13^C and ^1^H NMR spectroscopy. In ^1^H NMR, the methoxy protons resonance singlet at 3.64 ppm confirms the presence of methyl esters in *E. sativa* biodiesel (Figure 7). The appearance of methoxy proton and CH_2_ proton are the peaks in biodiesel for the confirmation of methyl esters. The peak present at 0.84 ppm represents terminal CH_3_ proton, an intense signal at 1.25 ppm represents CH_2_ proton of carbon chain, and signals at 1.55 ppm and 5.3 ppm are related to β-carbonyl methylene protons and olefinic hydrogen, respectively. Similar ^1^H NMR peaks in *E. sativa* biodiesel were observed by Shah et al.^86^ and Tariq et al^75^. The spectrum for ^13^C NMR of *E. sativa* oil biodiesel is presented in Figure 8. The resonance signals at 51.3 ppm and 174 ppm indicating ester carbonyl (-COO-) and C-O, respectively, confirms the conversion of *E. sativa* oil to biodiesel. Peak in the range of 127-131 ppm attributes to sp^2^ hybridized carbons of olefinic double bonds, showing unsaturation methyl eaters. The peaks around 22-34 ppm represent C-C bonds in fatty acid chains, indicating the presence of long chain fatty acid in *E. sativa* oil biodiesel. Similar peaks in *E. sativa* methyl esters were found by Tariq et al.^75^ and Shah et al.^86^. It is noteworthy that the NMR peaks across all treatments remained consistent, indicating that CuS NPs and CuSO_4_ treatment did not induce significant alteration in the biodiesels structure and resulting performance.

## Conclusion

The study indicates the substantial role of CuS NPs in improving plant growth, yield and biochemical attributes under field condition. The soil application of NPs enhanced agronomic parameters, oil percentage and antioxidant activities. Linseed oil being a feedstock for biodiesel, the improved oil percentage ultimately leads to enhanced biodiesel production. Comprehensive FT-IR, NMR, and GC-MS analysis of the biodiesel synthesized from oil extracted from treated and untreated plant seeds did not show significant alteration in composition. Moreover, fuel properties lie within international standard for biodiesel, demonstrating the feasibility of biodiesel produced from CuSO_4_ and CuS NPs treated plants. These outcomes highlight the dual importance of CuS NPs in agriculture and biofuel production and offer a sustainable approach to agricultural practices and renewable energy production. However, further research is needed to evaluate the long term ecological and environmental impact of metallic nanoparticles application in agriculture. Although, CuS NPs improved various plant parameters, it is important to evaluate the possible risk associated with NPs application in the cultivation of plants. The long run effect of NPs on the environment needs to be evaluated and further studies may be carried out on NPs accumulation in the soil and water. From literature, it has been ascertained that NPs may remain in the environment and affect the soil microbiota and productivity of crop. The present study may be considered as optimal, yet further studies need to be carried out in order to find that NPs application in agriculture is safer for food chain.

## Data Availability

All data generated or analysed and materials used during this study are included in this article, further inquiries can be directed to the corresponding author.
